# Halochromic Polystyrene Nanofibers Obtained by Solution Blow Spinning for Wine pH Sensing

**DOI:** 10.3390/s20020417

**Published:** 2020-01-11

**Authors:** Kelvi W.E. Miranda, Caio V. L. Natarelli, Adriana C. Thomazi, Guilherme M. D. Ferreira, Maryana M. Frota, Maria do Socorro R. Bastos, Luiz H. C. Mattoso, Juliano E. Oliveira

**Affiliations:** 1 Graduate Program in Biomaterials Engineering, Federal University of Lavras, Lavras 37200-000, Brazilcaionatarelli@poli.ufrj.br (C.V.L.N.); 2 Nanotechnology National Laboratory for Agriculture, Embrapa Instrumentação, São Carlos 13560-970, Brazil; adriana.thomazi@embrapa.br (A.C.T.); luiz.mattoso@embrapa.br (L.H.C.M.); 3 Department of Chemistry, Federal University of Lavras, Lavras 37200-000, Brazil; guilherme.ferreira@dqi.ufla.br; 4 Food Engineering Department, Federal University of Ceara, Fortaleza 60356-000, Brazil; mary.m.frota@gmail.com; 5 Packaging Laboratory, Embrapa Agroindustria Tropical, Fortaleza 60511-110, Brazil; socorro.bastos@embrapa.br; 6 Department of Engineering, Federal University of Lavras, Lavras 37200-000, Brazil

**Keywords:** nanofiber, sensor, smart packaging

## Abstract

Colorimetric sensors developed by the solution blow spinning (SBS) technique have a rapid response to a variation in different physicochemical properties. In this study, polystyrene nanofibrous (PSNF) mats containing the bromothymol blue (BTB) indicator were obtained by SBS for the pH sensing of wine sample. The incorporation of the indicator did not promote changes in fiber diameter but led to the appearance of beads, allowing for the encapsulation of BTB. The halochromic property of BTB was retained in the PSNF material, and the migration tests showed that the indicator mats presented values below the maximum acceptable limit (10 mg dm^−2^) established by EU Commission Regulation No. 10/2011 for foods with an alcohol content up to 20%. The present study opens the possibility of applying nanostructured materials to innovative food packaging which, through nanosensory zones, change color as a function of the food pH.

## 1. Introduction

The engineering of nanostructured materials has played an important role in the development of techniques designed to obtain nanomaterials with great potential for application in biomedicine [1], civil construction [2], cosmetics [3], and agribusiness [4] among others. In particular, the food industry has benefited from technologies involving nanomaterials [5,6], including the development of active and intelligent packaging [7,8] that has been commercially adopted as a solution for quality control and the shelf-life extension of foods [7,9] and beverages [10], for example, sensible to changes in the pH of aqueous media [11] and vapor [12].

Intelligent packaging incorporates devices that are able to monitor, in real time, the quality of the food and/or the packaging itself through responses to chemical changes, such as variations in pH [7,13,14], CO_2_ production [15], food freshness [16], or a time–temperature indicator [17], among others. This set of chemical modifications can be evaluated by the appropriate use of polymer nanomaterials in the construction of sensors. However, to generate polymer nanomaterials with specific properties amenable to intelligent packaging, techniques are needed that can accurately modulate the structure, size and composition of materials on a nanometric scale and allow their proper interaction/mixing.

The use of colorimetric sensors is one of the most basic and convenient analytical techniques, being easy and low-cost [18]. The technique allows to identify changes in the surrounding environment without requiring sophisticated equipment and trained manipulators [19]. The basis of colorimetric detection is the analysis of the color change caused by the generation of colored compounds (chromophore) from the reaction between the chromogenic reagent and the analyte [18,19].

Among several advantages, colorimetric sensors are attractive and are used for identification of analytes in different areas [20], such as detecting ammonia in aqueous solution or in a gas phase [19], urea in biological solutions [21], biochemical changes in sweat [22], conformational changes in proteins [18], volatile amines released during the deteriorating state of fish [23], ferric ions [24], and discrimination against organophosphate pesticides [25].

Polymer filaments can be produced in two ways by electrospinning (ES) and solution blow spinning (SBS). From these methods, it is possible to obtain fibers of the sub-, micro-, and nanometric scale [26]. However, the ES technique requires the use of high voltage, the use of solvents with medium dielectric properties and has a small-scale production [27,28]. In contrast, the SBS method is considered safe, low cost and has a high production rate, significantly higher than electrospinning [26,29,30].

In the context of polymer nanomaterials, the SBS technique is considered an innovation in obtaining versatile one-dimensional nanomaterials, especially nanofibers. In this technique, two parallel concentric fluid streams: (i) a polymer solution formed by a volatile solvent; and (ii) a pressurized gas (O_2_ or N_2_) move in a concentric and parallel manner [29,31]. When the solvent evaporates, the nanometric fibers are formed. This type of nanomaterial can exhibit a large surface area and a high porosity depending on the choice of polymer [32] and solvent, providing a multifunctional characteristic for the application of these nanomaterials as sensors [30].

The majority of the research involving nanofibers has focused on the development of materials for areas such as tissue engineering, catalysis and chemical sensors, highlighting mainly the use of the ES technique [33,34,35]. In the food area, such applications are still considered innovative, particularly the immobilization of indicator substances in nanofibers that can lead to the development of halochromic sensors. Agarwal et al. [27], for example, showed that the incorporation of different combinations of indicator dyes (methyl red, bromocresol green, bromothymol blue (BTB), phenol red and phenolphthalein) in the formation of nylon-6 fibers by ES did not modify the fiber morphology nor the halochromic profile of the indicator. Schoolaert et al. [36] obtained colorimetric sensors from electrospun nanofibers of a polymer blend (polycaprolactone/chitosan) incorporated with methyl red and rose bengal. The nanofibrous mats quickly responded to the pH change. More recently, the SBS technique has been utilized for the development of indicator nanofibers. Khattab et al. [11] developed a mat with a reversible halochromic potential; the mat was formed with polyacrylonitrile (PAN) and tricyanofuran-hydrazone (TCF-H) via the SBS technique, yielding a sensitivity to modifications in the medium (vapor or aqueous) pH. The authors proposed the use of the PAN-TCF-H halochromic nanofibrous sensor for the detection of amines and ammonia. Studies such as this open the door for applications of intelligent packaging, given that biogenic amines (histamine, cadaverine, tyramine and tryptamine) can promote food poisoning [37].

In the present study, the SBS technique was used to obtain polystyrene (PS) halochromic nanofibers integrated with the BTB indicator. The PS/BTB nanofibrous mats were investigated with respect to fiber morphology, indicator migration (dye leaching process) in aqueous media and halochromic behavior in aqueous media at different pH and exposed to acid vapors in red wine samples.

## 2. Experimental

### 2.1. Materials

Polystyrene (PS) with a molar mass of 1.9 × 10^5^ g mol^−1^ was purchased from Sigma-Aldrich (St. Louis, MO, USA) and used to obtain the nanofibers. Toluene (ACS reagent grade, Labsynth^®^, São Paulo, SP, Brazil) and acetone p.a. (ACS solventes, LabSynth^®^,São Paulo, SP, Brazil) were used as spinning and solubilization indicators, respectively. Bromothymol blue, BTB, (Qhemis, São Paulo, SP, Brazil) was incorporated into the nanofibers to obtain the indicator mats. Phosphoric acid (ACS solventes, Labsynth^®^, São Paulo, SP, Brazil), monobasic sodium phosphate (Dinâmica Ltd.a., São Paulo, SP, Brazil), dibasic sodium phosphate (Cromoline Química Fina Ltda., São Paulo, SP, Brazil), anhydrous sodium carbonate (LabSynth^®^, São Paulo, SP, Brazil), sodium bicarbonate (LabSynth^®^, São Paulo, SP, Brazil) and sodium hydroxide (LabSynth^®^, São Paulo, SP, Brazil) were used in the preparation of buffer solutions and for pH adjustments. The halochromic profile of the mat was studied in the presence of the following acids: glacial acetic acid P.A. (Dinâmica^®^, São Paulo, SP, Brazil) (AA), hydrochloric acid P.A. (ACS Dinâmica^®^, São Paulo, SP, Brazil) (HCA) and sulfuric acid P.A. (ACS LabSynth^®^, São Paulo, SP, Brazil) (SA). For the beverage test, a Merlot dry red wine (12.5% alcohol content) obtained from a local supermarket was used.

### 2.2. Solution Blow Spinning (SBS)

The polystyrene nanofibrous (PSNF) mats and PSNF mats with the pH indicator (PSNF/BTB) were obtained by the SBS technique. A 30 wt.% PS solution was prepared in toluene, while solutions with different concentrations of BTB (0.05%, 0.1%, and 0.2%, dry weight) were prepared in acetone. The BTB solution pH was adjusted to 13 with a 1 mol L^−1^ NaOH solution. All solutions were prepared at room temperature (25 °C) while stirring at 500 rpm. Next, the PS and BTB solutions were blended at a ratio of 3:1 for subsequent spinning at room temperature (25 °C). PS nanofibers with BTB contents of 0.05, 0.1 and 0.2%, dry weight was prepared and called PSNF/BTB-0.05, PSNF/BTB-0.1, and PSNF/BTB-0.2, respectively. Control nanofibers (PSNF control) were obtained using the same procedure in the absence of BTB.

**Figure 1:**
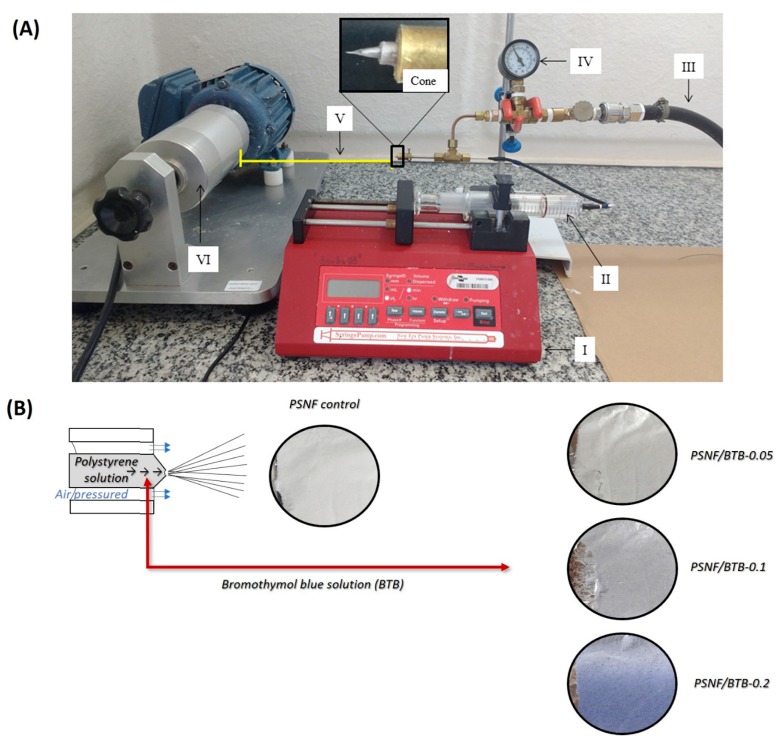
(**A**) Representation of the solution blow spinning (SBS) system for obtaining fibers: (I) controlled ejection pump; (II) polymeric solution to be ejected; (III) pressurized gas piping; (IV) gas pressure gauge; (V) working distance for solvent evaporation; and (VI) rotary collector; (**B**) Polystyrene nanofibrous mats with different bromothymol blue concentration (0.05, 0.1 and 0.2% (*w*/*w*)), obtained by SBS technique with collection time of 30 min at room temperature and RH ≤ 55%.

The blow spinning process apparatus is represented in Figure 1A. The process was performed using an air compressor (Schulz, model 10VL/200-2HP, Santa Catarina, Brazil) with 100 kPa moisture-free gas. In addition, an injection pump (NE-300, New Era Pump Systems, New York, NY, USA) equipped with a glass syringe (F-5500-A, Ismatec, Wertheim, Germany) was used. The SBS system operated at an ejection rate of 7 mL h^−1^ through a 0.5 mm diameter stainless-steel needle. To promote fiber formation during the ejection process, a needle protrusion distance of 0.2 mm was used and a working distance of 15 cm (distance between the needle and the rotating collector). The fibers were gathered on the surface of a collector rotating at 420 rpm for 30 min. Room temperature and relative humidity (RH) were 25 °C and RH ≤ 55%, respectively. The samples were placed in a desiccator under vacuum for 3 days for prior subsequent analysis.

### 2.3. Characterization of the Nanofibers

The nanofibrous mats were characterized by Fourier transform infrared (FTIR) spectrophotometry using a spectrophotometer model Vertex 70 Bruker (Ettlingen, Germany). The spectra were recorded in attenuated total reflectance (ATR) mode, accumulating 32 scans over a spectral range of 4000 to 400 cm^−1^ at a resolution of 4 cm^−1^. The fiber characterization was performed before and after 24 h of material immersion in solutions at pH = 2.0, 6.8 and 10.7. In addition to the visual color change of the nanofibrous mat, these pH values were determined through preliminary analyses to serve as reference values for evaluating the degradation of various foods.

Scanning electron microscopy (SEM) analyses were performed using a Zeiss DSM 960 (Jena, Germany) microscope. The samples were previously coated with gold using an SCD 050 Bal-Tec sputter coater (Balzers Union AG, Balzers, Liechtenstein). The mean fiber diameters were calculated using ImageJ software (National Institutes of Health, Bethesda, MD, USA), analyzing the morphology of 100 randomly chosen fibers.

### 2.4. Study of BTB Migration and Colorimetric Analysis

The migration assays followed the methodology proposed by Agarwal et al. [27] with modifications to determine the transfer of BTB bound to the PSNF mat to aqueous media after immersion. The assays were performed by immersing approximately 380 mm² of PSNF/BTB-0.2 mat in 100 mL of food simulator solutions B and C (3% (*w*/*v*) AA and 20% (*v*/*v*) ethanol, respectively) according to EU Commission Regulation n. No. 10/2011 [38]. The absorbance of the supernatant solutions at 430 nm was determined in a UV-vis spectrophotometer (model UV-M51, BEL Engineering, Italy). All assays were performed at steady state at 25 °C.

The colorimetric analysis of the PSNF/BTB mats was performed by determining the color space coordinates of the system defined by the International Commission on Illumination (CIE), as obtained using a Minolta Chromameter CR-400 (Konica Minolta Sensing, Inc., Osaka, Japan). In the CIE color space: L*a*b*, L* stands for lightness, a* represents the position between green (negative value) and red (positive value) and b* the position between yellow (positive value) and blue (negative value). In addition, the hue angle (0–360°) was evaluated to identify and quantify the changes in the color of the sensor mat (a visual attribute) and the location of the color on the colorimetric circle. Mat color measurements were performed after 24 h of nanomaterial immersion in buffer solutions, and an average of ten readings was made for each analysis. The total color difference (Δ*E*) between two PSNF/BTB samples subjected to the different conditions was calculated by the equation:

(1) $$∆E\;=\;\sqrt[{2}]{\left[{\left(∆L^{*}\right)}^{2}+{\left(∆a^{*}\right)}^{2}+{\left(∆b^{*}\right)}^{2}\right]}$$

where ΔL* is the difference in lightness between the samples, while Δa* and Δb* are the differences in the red and yellow colors, respectively, between the samples.

### 2.5. Halochromic Evaluation of PSNF/BTB-0.2 Mats in Wine Samples

The halochromic response of the PSNF/BTB-0.2 mat to acid vapors generated in wine samples was evaluated. For that purpose, 10 mL of the wine sample was added to test tubes doped with different acids: acetic acid P.A. (AA), hydrochloric acid P.A. (HCA) and sulfuric acid P.A. (SA) at the following concentrations: 0.0, 1.2, 2.4, 4.8, 7.6 and 10.0 gL^−1^, according to methodologies adapted from Lozano et al. [39] and Hopfer et al. [40]. Then, a PSNF/BTB-0.2 sensor mat, with a diameter of 1 cm², was fixed on the inside of each tube lid, which was then closed quickly and left to stand at a temperature of 25 °C. For the liquid-vapor equilibrium to be reached, a time of 30 min was used, as established in the previous analyses. After 30 min of contact, the mats were removed and immediately analyzed by a Minolta Chromameter CR-400, as described in Section 2.4.

### 2.6. Sensitivity and Limit of Detection (LOD)

The sensitivity of the nanofiber sensor to determine the presence of acid vapors was determined by the slope of the analytical curve (α) in the graph of ∆E versus acid concentration. The LOD was calculated using the following equation [41,42]:

(2) $$LOD\;=\;\frac{3\times Sb}{\alpha }$$

where *S_b_* corresponds to the relative standard deviation of the blank.

## 3. Results and Discussion

### 3.1. Morphological and Structural Characterization of Nanofibrous Mats

The PSNF mats containing different BTB concentrations, obtained by the SBS technique, are shown in Figure 1.

**Figure 2:**
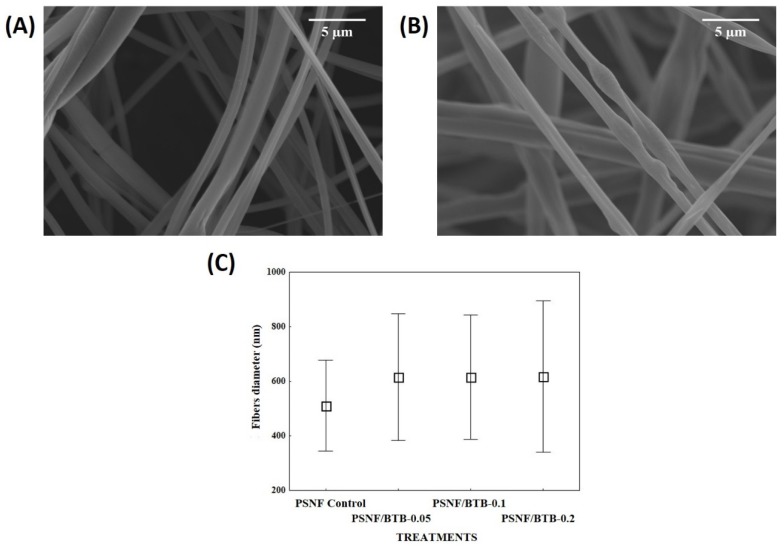
SEM micrographs of PSNF obtained by SBS: (**A**) polystyrene nanofibrous (PSNF) control; (**B**) PSNF/BTB-0.2; and, (**C**) average nanofiber diameters as a function of different BTB concentrations (mean ± standard deviation; n = 100).

The increase in the BTB concentration in the spinning solution resulted in a visual change in the color of the nanofibrous mats from white to blue, and for the highest BTB concentration (PSNF/BTB-0.2), a higher intensity of the blue color was observed. To evaluate the effect of adding the indicator on the morphological profile of PSNF mats, SEM micrographs in the absence and presence of BTB were obtained, and the respective mean diameters were calculated, as shown in Figure 2.

The morphological profiles of nanofibrous mats in the absence and presence of BTB were similar. The micrographs showed, however, that the presence of BTB at a concentration of 0.2% m/m (Figure 2B) led to the formation of imperfections in the morphology of polymeric fibers. Nevertheless, the obtained fibers did not show differences in mean diameter for the different indicator concentrations used (Figure 2C). While the PSNF control showed a variation in the diameters of the nanofibers between 344 and 677 nm, the PSNF/BTB-0.2 diameters ranged between 340 and 895 nm.

According to the literature, the use of pH indicators to obtain polymeric nanofibers usually did not result in changes in the mean diameter of the fibers or the formation of beads during spinning [27,28]. However, the literature reported that the incorporation of indicators may promote the formation of beads in the morphology of nanofibers. Agarwal et al. [27], for example, observed the presence of beads in nylon-6 nanofibrous mats containing a combination of methyl red, bromocresol green, BTB, red phenol and phenolphthalein indicators obtained by ES. Khattab et al. [11] observed in the morphology of PAN nanofibers that an increase in the concentration of the indicator, 5% of TCF-H, promoted the formation of beads, indicating the presence of the dye in the polymer matrix. The formation of beads can be due to two situations: (i) adjustments in the blow spinning parameters, such as gas pressure and polymer solution injection rate [26,29]; and/or (ii) instabilities of the polymer jet ejected while spinning due to the high concentration of the indicator used. Thus, PSNF mats may have acted as reservoirs or encapsulators of the BTB indicator in PS fibers, as shown visually in Figure 1 by the intense blue color of the mat.

**Figure 3:**
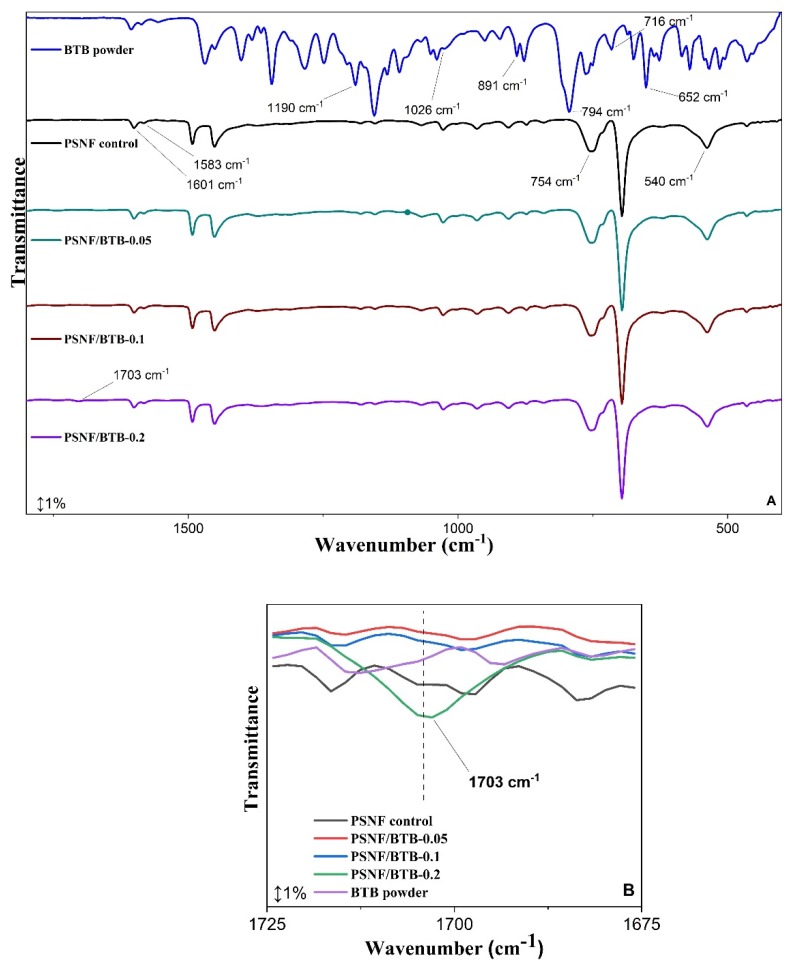
(**A**) FTIR spectra of PSNF control, NFPS/BTB nanofibrous mats at different indicator concentrations and pure BTB; and, (**B**) Wavelength spectra magnification at 1703 cm^−1^.

The addition of components to the polymer matrix during the spinning process for obtaining the nanofibers can promote changes to or the emergence of new bands in the FTIR spectra of the samples, indicating possible intermolecular interactions or chemical bonds between the components forming the nanofibrous mats [43]. Thus, to evaluate how the incorporation of the indicator occurred during the nanofiber formation process, infrared spectra of the nanofiber mats were obtained after drying, in the presence and absence of BTB, and compared with the spectra of pure PS and BTB (Figure 3).

The pure PS polymer spectrum (not shown) did not differ from the spectrum of the PS nanofiber in the absence of BTB, indicating that the blow spinning process for fiber formation did not affect the polymer structure. In the spectrum of the control PS nanofibers, the polymer was atactic. This characteristic was indicated by the bands at 754 and 540 cm^−1^ referring to the bending vibration and C−H out-of-plane bending vibration of aromatic rings, respectively [44,45,46,47]. The band at 1601 cm^−1^ was associated with the symmetric C=C in-plane vibration of the monosubstituted aromatic ring, and the one at 1583 cm^−1^ with the C=C in-plane stretching of the benzene ring [45,48,49]. The atactic nature of polystyrene (aPS) refers to its disordered, amorphous structure [47,49]. This feature favors the internal and external incorporation of BTB in the structure of the obtained fibers.

In the pure BTB spectrum, the characteristic bands were identified in regions near 1190 cm^−1^ and 1026 cm^−1^, corresponding to vibrations of the −SO_3_ group [50]. The bands at 891 and 794 cm^−1^ were associated with symmetric and asymmetric S−O−C stretching, respectively [51]. A low-intensity band associated with the C−S stretching vibration was observed at 716 cm^−1^ [52]. At 652 cm^−1^, a narrow band appeared due to the vibrational stretching of the aliphatic C−Br bond [50].

The addition of BTB to the PS fibers did not lead to the appearance of bands characteristic of the indicator in the spectra of the modified PSNF/BTB-0.05 and PSNF/BTB-0.1 fibers. Furthermore, the BTB incorporation process did not promote a displacement of bands associated with PS in the spectra of the modified fibers compared to those in the spectrum of the control PSNF. This result suggests that the indicator incorporation at the lowest concentrations may have occurred due to weak intermolecular interactions of London dispersion forces between the hydrophobic groups of the BTB (benzene rings and the −CH_3_ groups) and the hydrophobic surface of the polymer [53]. The presence of increasing amounts of one or more components in a sample may make a band in the infrared spectrum more evident [54]. However, the low concentrations of the indicator used may have resulted in the absence of bands associated with BTB in the PSNF/BTB-0.05 and PSNF/BTB-0.1 fiber spectra. Khattab et al. [11] also observed that the incorporation of the TCF-H indicator in PAN nanofibrous mats did not lead to the appearance of new spectral bands relative to the mat without the indicator, suggesting that there were no chemical reactions during the incorporation process. However, in the PSNF/BTB-0.2 spectrum, a new low-intensity band, absent both in the FTIR spectra of the other fibers and in the FTIR spectrum of pure BTB, appeared at 1703 cm^−1^ (Figure 3B). This band was most likely associated with the stretching of the C=O group of BTB [52], indicating that the indicator was in its deprotonated form, therefore, providing the blue color to the nanofibrous mat (Figure 1) with potential indicator action for pH sensing.

### 3.2. Analysis of the PSNF/BTB-0.2 pH Sensing Capacity by Immersion

The variation in the pH of a medium is an important quality indicator of food during storage because this parameter signifies the chemical alteration of food due to spoilage reactions [53,55,56]. In addition, different types of food may exhibit distinct variations in pH levels during spoilage. For example, low-acidity foods with pH between 4.5 and 7.0 (meat, fish and eggs) exhibit a high microbial spoilage activity, while spoilage in high-acidity foods (fruits, vegetables and beverages, such as wine) is mostly caused by fungal and biochemical activity. In this sense, the PSNF/BTB-0.2 indicator mat was evaluated for its pH sensing capacity in an aqueous medium.

**Figure 4:**
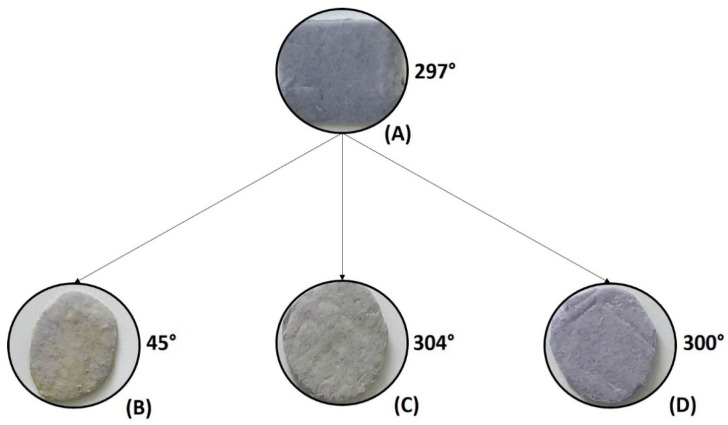
PSNF/BTB-0.2 mats obtained by SBS submerged for 24 h in buffer solutions with different pH values: (**A**) PSNF/BTB-0.2 (reference), without submerging, buffer solutions with pH (**B**) 2.0, (**C**) 6.8 and (**D**) 10.7. Hue angle values are indicated for each condition evaluated.

Samples of PSNF/BTB-0.2 were submerged in solutions with pH of 2.0, 6.8 and 10.7. Figure 4 shows the visual changes in the mat color, which were quantified by the hue angle. The total color difference (Δ*E*) between the samples analyzed is shown in Table 1.

The PSNF/BTB-0.2 (reference) mat, in contact with the acidic medium, showed a reversible change in color from blue to yellow, and the hue angle ranged from 297° to 45°. Furthermore, the Δ*E* values obtained were higher than 5 for all sample combinations, indicating that the difference between the colors of the mats subjected to different pH values can be easily identified with the naked eye [57]. Furthermore, the difference in color between mats subjected to acidic and basic environments was greater than 12, showing that the colors of the two materials belong to different color quadrants [27], that is, they ranged from quadrant I (0–90°) to quadrant IV (300–360°).

In addition, the ability of the PSNF/BTB-0.2 sensor to respond to pH change was compared with that of a sensor formed by a PS film incorporated with the same BTB concentration (Table 1). The Δ*E* values were lower for the films than those for the nanofibers. The PS/BTB-0.2 films presented acid-basic halochromic capacity smaller than 5, showing that the colors of both materials belong to the same quadrant [27]. These results indicate that PS films embedded in BTB are not adequate as sensor of pH, highlighting the benefits of using nanofiber mats with potential application in monitoring the shelf life of food products.

**Figure 5:**
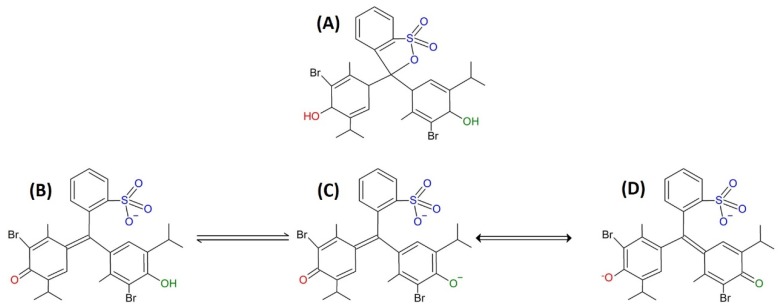
(**A**) Structure of the BTB molecule in: (**B**) acid medium, (**C**) basic medium and, (**D**) is a resonant structure of species C.

**Figure 6:**
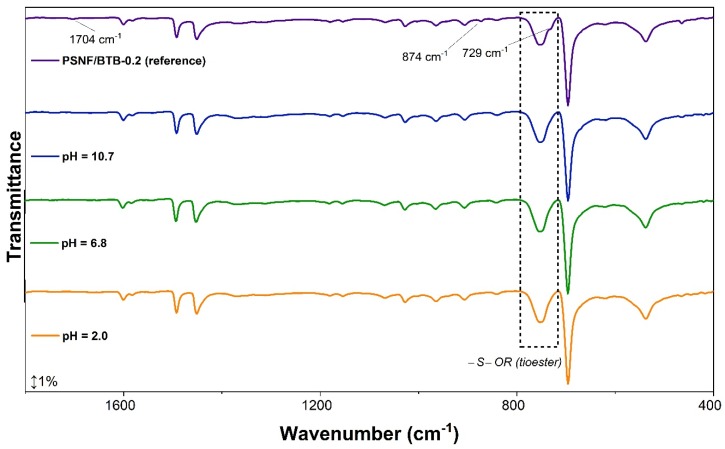
PSNF/BTB-0.2 infrared spectrum (reference) before and after 24 h immersion in pH 2.0, 6.8 and 10.7 solutions. The nanofibrous mats were dried in desiccated for 48 h.

BTB is a dye with a pK_a_ value of 7.1, and in acid media (pH < 6) is completely protonated, exhibiting a yellow color. In basic media (pH > 8), the dye is almost completely in its deprotonated form and has a blue color [55]. The BTB structures at different pH values are shown in Figure 5. The PSNF/BTB-0.2 mat color change from blue to yellow when added to acid media resulted from the protonation of the BTB molecules on the surface of the nanofibers. Interestingly, the color change of the PSNF/BTB was a reversible process, probably because of the hydrophobic incorporation that retained the ionizable group of the BTB free to be protonated/deprotonated. In basic media, the dye remained in its deprotonated form, maintaining the bluish color of the fibers. Figure 6 shows the FTIR spectra of the PSNF/BTB-0.2 fibers after immersion in aqueous solutions with different pH values.

The immersion of the PSNF/BTB-0.2 mat in the different aqueous media (regardless of the pH) led to the disappearance of some bands in the FTIR spectra including the band at 1703 cm^−1^, related to C=O stretching [52], and the bands between 900–700 cm^−1^, associated with the symmetric S−O−C (thioester) vibrational stretching of BTB [52,58,59]. Most likely, immersion in aqueous media led to the breakage of the O−C bond of the thioester (structure A in Figure 5) induced by the deprotonation of the hydroxyl group, leading to the disappearance of the band at 729 cm^−1^. This result may have been caused by changes in the structure of the indicator due to protonation/deprotonation of the sulfonate and phenyl groups [55].

**Figure 7:**
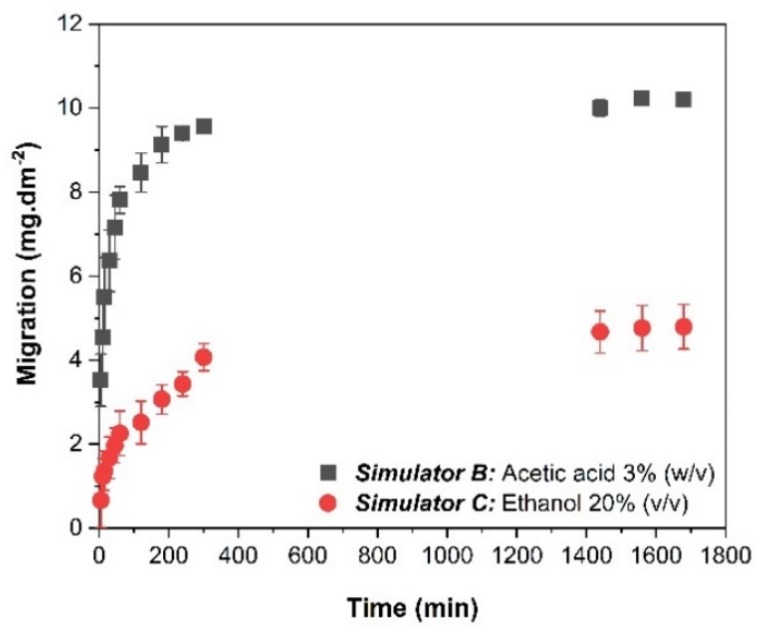
BTB migration from the reference mats (PSNF/BTB-0.2) in food simulators.

Despite the important changes observed in the FTIR spectra of the PSNF/BTB-0.2 mat after immersion in aqueous media with different pH values, the disappearance of some bands could be associated with the migration of the indicator from the fibers to the solution, reducing the concentration of the BTB in the indicator mat and making it undetectable in the FTIR spectrum. At the same time, this phenomenon of migration has great importance in determining the application of indicator mats with foods. Thus, the migration of BTB from the PSNF/BTB-0.2 mats was evaluated in food simulation media as a function of time, and the results are shown in Figure 7.

BTB migration from the mats occurred for the two simulators evaluated, and after 1440 min (24 h) of migration, the BTB phase equilibrium was reached for both simulators. At equilibrium, the migration to simulator B (3% (*w*/*v*) AA) was approximately twice that to simulator C (20% (*v*/*v*) ethanol), exceeding the maximum limit defined by the EU. Commission Regulation No 10/2011 (10 mg dm^−2^) [38]. Therefore, the application of the proposed system is not adequate for foods with a hydrophilic character at pH below 4.5. However, as the migration in simulator C reached equilibrium at approximately 5 mg dm^−2^, the proposed system can be applied with hydrophilic foods containing up to 20% alcohol content and possessing a significant amount of organic ingredients that make the food more lipophilic [38].

The high migration of the dye to simulator B can be explained by the lower stability of the PS-BTB interaction and/or the greater solubility of the compound when exposed to acidic media, thus, inducing the release of the BTB incorporated in the nanofibers.

### 3.3. Application of Halochromic PSNF/BTB-0.2 to Wine (Volatile Acidity)

The wine identity and quality standard (IQS) depends on the sugar content (smooth, dry, semidry, etc.), the type of grape (cabernet sauvignon, malbec, merlot, etc.) and the color (red, white or rosé), promoting a multifaceted definition of wine quality [39,40]. The loss of wine quality causes changes in the main sensory attributes (aroma and flavor), which can be quickly identified by the unpleasant odors formed by the volatile compounds produced during the fermentation process [39]. During the wine spoilage process, aromas are formed that are characteristic of ethyl acetate, cork-related compounds, sulfur, and mainly, AA [39].

Other acids are found in the food industry. HCA is widely used in the hydrolysis of starch and proteins [60]. SA, when highly diluted, can be used by the food industry as an acidulant, a food additive recognized by the number E513, in the dairy industry for the production of cheese [61] and in sugar factories to obtain ethanol [62]. In addition, the acid is used in the wine industry for the acidification of calcium tartrate to obtain free tartaric acid [63]. This usage explains the need to evaluate the response of the sensor to the vapors of systems formed by different acids.

**Figure 8:**
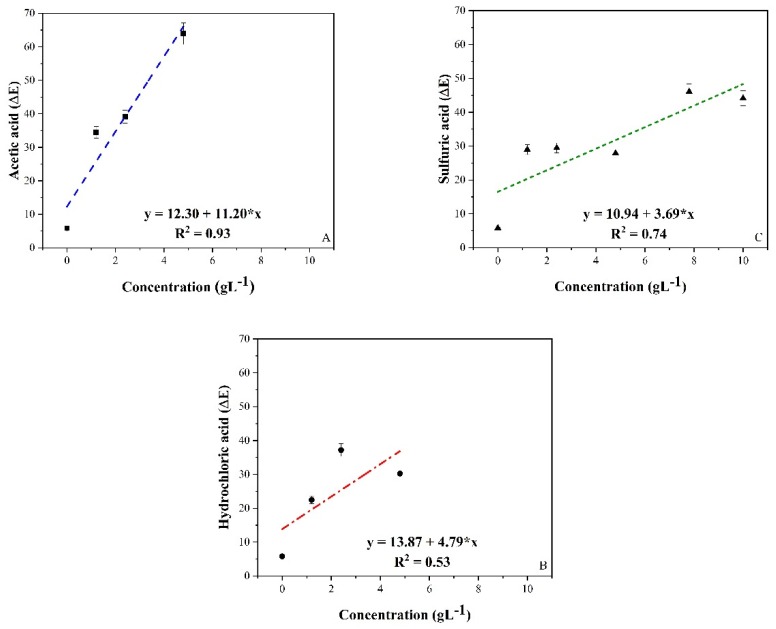
Colour variation (∆*E*) of PSNF/BTB-0.2 nanofibers mats subjected to acid vapors produced in doped wine samples with different acid concentrations (0, 1.2, 2.4, 4.8, 7.6 and 10.0 g L^−1^): (**A**) acetic acid, (**B**) hydrochloric acid and, (**C**) sulfuric acid, after 30 min of liquid-vapor equilibrium.

Figure 8 shows the halochromic potential of the PSNF/BTB-0.2 nanofibrous mat to the vapor of wine samples doped with different types of acids: AA, HCA and SA. The vapor analysis is necessary because in red wine samples, the very compounds that confer the red color to the beverage (anthocyanins) will mask the analysis of color changes.

The results show a high sensitivity of the nanofiber mat to the volatile acid compounds of the sample, indicating a color change from blue to yellow. This color change can be explained by the acid-base equilibrium discussed in Section 3.2 being displaced by the action of volatile acids on the indicator material encapsulated in the nanofibers.

The response of the sensor showed a better linearity for AA than for the inorganic acids. Furthermore, the sensitivity to AA vapors (11.20 L g^−1^) was greater than that observed for CA, and SA, with 4.79 and 3.79 L g^−1^, respectively. The LODs of the components were calculated, obtaining values of 0.53, 1.24 and 1.60 g L^−1^. Most likely, the method of detection used in our methodology, i.e., through contact between the mat and the vapor generated by the sample, favored the sensing of AA. This conclusion is due to AA being a volatile acid, unlike the others that have low vapor pressures when in aqueous solution. Most likely, in the case of inorganic acids, there was protonation of the most volatile wine compounds, which interacted with the fibers.

Mat color changes were observed by the naked eye at AA concentrations higher than 1.2 g L^−1^ (Figure 8A). The hue angle of the PSNF/BTB-0.2 (reference) mat changed from 300° (blue color) to a range from 82° to 95° (yellow color). For the analysis of volatile acids in the system formed with HCA (Figure 8B), the color change began at a concentration of 1.2 g L^−1^, with eventual changes from blue (300°) to a yellow range of 68° to 87° for the hue angle. For doping with SA (Figure 8C), the change in color was only perceptible at values greater than 4.8 g L^−1^, and the angle ranged from 81° to 89° for yellow.

According to the FAO (Food Administration and Organization of the United Nations) in collaboration with FAO’s Rural Infrastructure and Agro-Industries Division, wine quality is measured by chemical analysis, including the volatile acid content [64], which should be lower than 1.1 g L^−1^ for wines [64]. According to Brazilian law, table wines, fine wines, and noble wines may have up to 1.2 g L^−1^ of volatile acidity [65].

The material developed in this study demonstrates potential for use as a wine quality indicator through the color change and color intensity (hue angle) of a nanofibrous mat. The hue angle ranged from 341° (blue color) in the control mat to 82° (reference mat subjected to an AA concentration of 1.2 g L^−1^), increasing to 95° (reference mat subjected to an AA concentration of 10.0 g L^−1^).

## 4. Conclusions

The incorporation of the BTB pH indicator in PS nanofibers by the SBS technique was performed successfully. In this context, the SBS technique can be considered easy to use for the development of smart nanomaterials with staggered production profiles. The obtained smart nanofibers showed a visual sensitivity to changes in pH in aqueous and vapor media. The incorporation of BTB into the PS fibers did not promote intense modifications to the morphology of the nanofibers but led to the formation of beads that promoted the encapsulation of the dye. The mean PS fiber diameters showed no significant difference, regardless of the BTB concentration used. The migratory potential of the PSNF/BTB-0.2 was considered high for foods with pH < 4.5, but the values obtained in this study were within the limit established by law, especially for beverages with up to 20% alcohol content. Finally, the halochromic potential of the nanofibrous mat was correlated with a rapid chromatic response to red wine vapor, promoting a change in the color of the nanofibers due to the presence of volatile acids in the beverage. These results suggest that the PSNF/BTB-0.2 nanofibrous mats can be used as a nanomaterial sensor for multiple applications dealing with evaluation by direct surface contact or by indirect contact, e.g., through the measurement of volatile acids in the control of wine quality. This advancement opens a door for the use of halophytic nanofiber mats in intelligent packaging as quality sensors for food and beverage products that generate acid products during the spoilage stage.

## Figures and Tables

**Table 1:** Total color difference (Δ*E*) between PS/BTB-0.2: nanofibers and films submitted to different mediums: acid (pH 2.0), basic (pH 10.7) and neutral (pH 6.8).

Mediums Compared	Δ*E*	
	Nanofibers	Films
Acid—Basic	14.66	1.87
Acid—Neutral	8.26	5.30
Basic—Neutral	7.45	6.59
